# Evaluation of Immunogenicity and Efficacy of *Fasciola hepatica* Tetraspanin 2 (TSP2) Fused to *E. coli* Heat-Labile Enterotoxin B Subunit LTB Adjuvant Following Intranasal Vaccination of Cattle

**DOI:** 10.3390/vaccines9111213

**Published:** 2021-10-20

**Authors:** Gemma Zerna, Vignesh A. Rathinasamy, Hayley Toet, Glenn Anderson, Robert Dempster, Terry W. Spithill, Travis Beddoe

**Affiliations:** 1 Department of Animal, Plant and Soil Sciences and Centre for AgriBioscience, La Trobe University, Bundoora, VIC 3083, Australia; gmzerna@students.latrobe.edu.au (G.Z.); Vignesh.ambothirathinasamy@jcu.edu.au (V.A.R.); H.Toet@latrobe.edu.au (H.T.); T.spithill@latrobe.edu.au (T.W.S.); 2 Intensive Livestock, Fisheries & Aquaculture Research, Development & Extension, Department of Agriculture and Fisheries, Dutton Park, QLD 4102, Australia; glenn.anderson23@bigpond.com; 3 Virbac (Australia) Pty Ltd., Milperra, NSW 2214, Australia; robert.dempster@virbac.com.au

**Keywords:** vaccine, *Fasciola hepatica*, LTB, AB_5_ toxin, tetraspanin, tegument membrane, mucosal immunity, cattle

## Abstract

Fasciolosis, caused by the liver flukes *Fasciola hepatica* and *F. gigantica,* is an economically important and globally distributed zoonotic disease. Liver fluke infections in livestock cause significant losses in production and are of particular concern to regions where drug resistance is emerging. Antigens of the *F. hepatica* surface tegument represent promising vaccine candidates for controlling this disease. Tetraspanins are integral tegumental antigens that have shown partial protection as vaccine candidates against other trematode species. The *Escherichia coli* heat-labile enterotoxin’s B subunit (LTB) is a potent mucosal adjuvant capable of inducing an immune response to fused antigens. This study investigates the potential of *F. hepatica* tetraspanin 2 extracellular loop 2 (rFhTSP2) as a protective vaccine antigen and determines if fusion of FhTSP2 to LTB can enhance protection in cattle. Cattle were immunised subcutaneously with rFhTSP2 mixed in the Freund’s adjuvant and intranasally with rLTB-FhTSP2 in saline, accounting for equal molar ratios of tetraspanin in both groups. Vaccination with rFhTSP2 stimulated a strong specific serum IgG response, whereas there was no significant serum IgG response following rLTB-FhTSP2 intranasal vaccination. There was no substantial antigen specific serum IgA generated in all groups across the trial. Contrastingly, after the fluke challenge, a rise in antigen specific saliva IgA was observed in both vaccination groups on Day 42, with the rLTB-FhTSP2 vaccination group showing significant mucosal IgA production at Day 84. However, neither vaccine group showed a significant reduction of fluke burden nor faecal egg output. These results suggest that intranasal vaccination with rLTB-FhTSP2 does elicit a humoral mucosal response but further work is needed to evaluate if mucosal delivery of liver fluke antigens fused to LTB is a viable vaccine strategy.

## 1. Introduction

Fasciolosis is a globally distributed zoonotic disease caused by the liver flukes *Fasciola hepatica* and *F. gigantica*, which infect a wide range of mammals including domestic ruminants. Substantial economic losses to the livestock industry occur from reduced quality and quantity of milk, meat, or wool in infected hosts (cattle, sheep, pigs, and goats) along with treatment costs [1,2,3,4]. Liver fluke infections are primarily treated with triclabendazole (TCBZ); however, the heavy reliance on TCBZ has led to the selection of drug resistant flukes in Australia, New Zealand, South America, Europe, and the UK [5,6,7,8]. Fasciolosis also affects over 2 million humans, with TCBZ resistant human cases seen in the Netherlands, Chile, and Turkey [5,7,8]. Sustainable and economically beneficial control of liver fluke infection requires new and effective vaccines [2,9].

Current vaccine candidates predominantly sit at the host–parasite interface with fluke excretory/secretory (E/S) molecules representing the lead *F. hepatica* vaccine candidates. These candidates, such as glutathione *S*-transferase (GST) and cathepsin L1 (CatL) protease, are yet to achieve substantial and reproducible protection with mean efficacies of 43% and 48% in cattle, respectively [10,11]. For commercialisation, it is generally accepted that a liver fluke vaccine should achieve 60–70% efficacy in cattle [12]. Considering E/S based vaccines fall below this threshold, it was proposed that the fluke’s outer tegument layer could contain protective molecules [9,12]. The tegument or surface tissue layer of flukes has direct contact with the host immune system and could contain antigens that stimulate the previously described potential immune-mediated killing mechanism, antibody dependent cellular cytotoxicity (ADCC), that is observed in rats, sheep, and cattle [9,12,13].

A key family of integral membrane proteins called tetraspanins (TSPs) have been identified in the adult *F. hepatica* tegument proteome, and several sequences encoding TSPs are observed within the fluke genome [9,14,15,16]. TSPs contain four transmembrane domains that anchor two extracellular loops with the second extracellular loop (ECL2) larger than the first (ECL1) and responsible for protein–protein interactions [17,18]. Several TSPs have been found through proteomic studies to be surface exposed or membrane bound in other trematodes species such as, *Schistosoma mansoni, S. japonicum, Opisthorchis viverrini,* and *Clonorchis sinensis* [19,20,21,22,23,24]. Recombinant ECL2 of SmTSP2 is a promising vaccine candidate against *S. mansoni,* achieving significant efficacies of 25–58% in vaccine studies in mice using different fusion molecules, and this molecule has now entered human clinical trials [25,26,27,28,29]. It has been identified that SmTSP2 is surface-exposed through immunofluorescence analysis and is strongly recognised by protective IgG1 and IgG3 antibodies from humans resistant to schistosomiasis [25,27]. Additionally, vaccination of hamsters with the *O. viverrini* tetraspanin (OvTSP2), an orthologue of SmTSP2, has also been found to significantly reduce the parasite burden by 27–34% compared to adjuvant controls: moreover, sera containing antibodies to OvTSP2 can block parasite extracellular vesicle (EV) uptake into biliary epithelial cells, highlighting one of the several unexplored functions of TSPs in fluke biology [30,31]. The *F. hepatica* TSP2 homologue could therefore be considered a potential vaccine candidate. Our laboratory has identified an orthologue of SmTSP2, termed here FhTSP2, that encodes a protein identified in the membrane of the adult liver fluke tegument proteome as well as in *F. hepatica* extracellular vesicles; interestingly, expression of FhTSP2 is upregulated early after parasite infection in the newly excysted juvenile (NEJ) life stage, which is the key target for a fluke vaccine [14,16,32].

Like other helminths, the liver fluke’s immunomodulatory behaviour is characterised by a potent type 2 helper T cell (Th2) immune response, with an active suppression of the host Th1 immune response against the fluke [33,34]. The pro-inflammatory host Th1 immune response has been associated with protection as it is the dominant immune response in Indonesian thin-tail (ITT) sheep, which are resistant to *F. gigantica* [35,36]. ITT sheep are naturally resistant to *F. gigantica* infections, showing higher levels of Th1-associated cytokines and specific IgG2 levels during infection [36]. Induction of the Th1 immune response or limiting the fluke induced Th2 response through an immunomodulating adjuvant could be imperative to gain an efficacious vaccine against liver fluke.

The AB_5_ toxin family are immunomodulatory protein adjuvants capable of binding to host cellular glycan receptors [37,38]. The *Escherichia coli* heat-labile enterotoxin (LT) and the cholera toxin (CT) produced by *Vibrio cholerae* consist of an enzymatically active A subunit and a homo-pentameric B subunit [37,39]. The toxic properties are derived from the A subunit, leaving the B subunit non-toxic and responsible for receptor binding and toxin internalisation [39]. The immunomodulatory properties of the B subunits differ between the toxins: in mice, CTB induces a polarising Th2 immune response and LTB induces a mixed Th1/Th2 immune response that can be manipulated through the route of administration [38]. Mucosal administration routes of LTB preferentially induce a Th1-like immune response, with parenteral routes stimulating a Th2-biased immune response [38,40]. Adjuvanticity can be triggered from the ring-like pentamer structure binding to ganglioside receptors, with LTB showing strong affinity towards ganglioside-GM1 [37,41]. Mucosal delivery of LTB is capable of inducing both systemic and mucosal immunity and can act as a carrier vehicle by increasing the immunogenicity towards antigens fused to LTB [42,43]. However, there are minimal investigations into the effects of LTB administrated intranasally in livestock.

In an attempt to boost the protective properties of any liver fluke vaccine, we hypothesised that liver fluke antigens could be fused with the LTB adjuvant in an attempt to drive the desired Th1 immune response. This study investigates the vaccine potential of *F. hepatica* TSP2 both unfused and fused to LTB. This study aimed to produce a fusion protein (rLTB-FhTSP2) and intranasally vaccinate cattle to test vaccine efficacy of a mucosal vaccine compared to rFhTSP2 delivered by subcutaneous vaccination. 

## 2. Materials and Methods

### 2.1. Integration of Constructs into Pichia pastoris

The mature gene for the human variant LTB (NCBI accession no. M178974) was mutated at the nucleotide site 274 (A to a G base) to replace the translated threonine at position 92 to alanine, removing the potential N-glycosylation site. The full-length *F. hepatica* TSP2 sequence was obtained from a previously described [44] in-house cDNA database (FhD #47871) (Supplementary Data S1) and used to design the expression construct containing the ECL2 domain (amino acid position 108 to 185). 

The FhTSP2 ECL2 sequence (termed rFhTSP2 here) was cloned after RNA isolation from adult *F. hepatica* collected from naturally infected sheep, as completed by [45]. Total RNA was isolated using the Pure link RNA mini kit (Invitrogen, Waltham, MA, USA), and contaminating DNA was removed by Turbo DNase (Ambion, Austin, TX, USA) according to the manufacturer’s instructions. Subsequently, the initial strand of cDNA was synthesised from total DNA-free RNA with oligo dT_(18)_ primers using the Tetro cDNA synthesis kit (Bioline, Eveleigh, NSW, Australia), and the full length open reading frame of the FhTSP2 sequence was amplified using forward (5′-TTGTAGCAGACTTGCCCGT-3′) and reverse (5′-ATGTGAATGTGGACCTGCTAG-3′) primers designed with the Oligo 7 program. Amplification occurred using the proofreading DNA polymerase, Velocity polymerase (Bioline), at an annealing temperature of 58 °C and visualised by agarose gel electrophoresis. The amplicon was purified from the gel with the Isolate II PCR and gel kit (Bioline) using the manufactuer’s instructions. Purified amplicons were processed using IMMOLASE^TM^ DNA polymerase (Bioline) to add the 3′ A overhangs for insertion into the pGEM T-easy vector system (Promega, Madison, WI, USA). Following transformation into DH5α *E. coli* cells, the plasmid containing the full-length FhTSP2 sequence was isolated using the Isolate II plasmid mini kit (Bioline) as described by the manufacturer. To generate an expression vector containing the ECL2 of TSP2, the plasmid containing the full-length sequence was amplified using ECL2 specific forward (5′-GGCGCGAATTCCTGAAGGACAAAGTCAAAGAGTAC-3′) and reverse (5′-GGCCGTCTAGATGTACGCAGCCGTCGGAGTAA-3′) primers at an annealing temperature of 60 °C with the Velocity polymerase (Bioline). Restriction enzyme sites, *EcoRI* (Promega) and *XbaI* (Promega) were included in the ECL2 amplification primers for digestion and ligation into the secretory expression *Pichia pastoris* pPICZαA vector (Invitrogen). 

Constructs encoding rLTB and rLTB-FhTSP2 were codon-optimised and commercially synthesised by Bioneer Pacific (Daejeon, South Korea), then cloned into the secretory *P. pastoris* pPICZαA vector (Invitrogen). For rLTB-FhTSP2 design, the N-terminus of the FhTSP2 ECL2 sequence was joined to the C-terminus of LTB via a short flexible linker sequence (GGGGS)_2_. A C-terminal polyhistidine tag (His tag) and stop codon were added upstream of the vector’s *c-myc* epitope and stop codon for both the rLTB-FhTSP2 and rLTB sequences. The unfused rFhTSP2 construct utilised the plasmid stop codon site, translating the vector’s his-tag and a *c-myc* epitope (Figure S1). 

All expression vectors were transformed into DH5α *E. coli* cells according to standard heat shock *E. coli* transformation protocols. Colonies that grew on low-salt LB agar plates (1% (*w*/*v*) tryptone, 0.5% (*w*/*v*) yeast extract, 0.5% (*w*/*v*) NaCl, 1% (*w*/*v*) agar, pH 7.5) containing 25 µg/mL of Zeocin were screened for the construct insert by AOX1 colony PCR according to the EasySelect^TM^ *Pichia* manual (Thermo Fisher Scientific, Scoresby, VIC, Australia). Recombinant clones were cultured overnight at 37 °C in a shaking incubator (250 rpm) in 10 mL of low-salt LB broth containing Zeocin (25 µg/mL), and plasmid from a single positive colony was isolated using the AccuPreps^®^ NanoPlus Plasmid Mini Extraction Kit (Bioneer Pacific), following the manufacturer’s instructions. 

Isolated plasmid DNA (~6 µg) was linearized using a *Sac*I (New England Biolabs, Ipswich, MA, USA) digest for optimal integration into the *P. pastoris* genome. Digestion was completed at 37 °C for 2 h in a buffer recommended by the manufacturer, and the enzyme was inactivated at 65 °C for 20 min. Linear plasmid DNA (1–1.5 µg) was transformed into chemically competent X33 *P. pastoris* cells, using the *Pichia* EasyComp^TM^ Transformation Kit (Thermo Fisher Scientific) according to the manufacturer’s instructions. Cells were then plated onto YPD agar (1% (*w*/*v*) yeast extract, 2% (*w*/*v*) veggietone peptone No. 1 (Oxoid Limited, Dublin, Ireland), 2% (*w*/*v*) dextrose, and 2% (*w*/*v*) agar) containing 100 µg/mL of Zeocin and incubated at 28 °C for 96 h. *P. pastoris* growth colonies were tested for the construct insert via AOX1 colony PCR with positive products analysed by nucleic acid sequence (AGRF, Melbourne, VIC, Australia). All DNA concentrations were estimated by NanoDrop 2000 spectrophotometer (Thermo Fisher Scientific). 

### 2.2. Expression and Purification of Recombinant Proteins

Positive yeast colonies were inoculated into 10 mL YPD seed-cultures (1% (*w*/*v*) yeast extract, 2% (*w*/*v*) veggietone peptone No. 1, 2% (*w*/*v*) dextrose, and 100 µg/mL Zeocin) and incubated for 72 h at 28 °C within a shaking incubator (200 rpm). Proteins were subsequently expressed in 400 mL BMMY medium (10 mL of seed-culture, 1% (*w*/*v*) yeast extract, 2% (*w*/*v*) veggietone peptone No. 1, 1% (*w*/*v*) yeast nitrogen base, 75 mM ammonium sulfate, 4 × 10^−5^% (*w*/*v*) biotin, 0.25% (*w*/*v*) dextrose, 100 mM KH_2_PO_4_ [pH 6], 100 µg/mL Zeocin, 0.5% (*v*/*v*) methanol, and two drops of antifoam 204) within a 2 L baffled flask. Cultures were incubated for 72–96 h at 28 °C with shaking (160 rpm). Methanol was added every 24 h to maintain a 0.5% (*v*/*v*) concentration for protein expression. Cultures were harvested at 4000× *g* for 30 min with supernatants transferred to dialysis tubing (12,400 Da MWCO, Sigma-Aldrich, St. Louis, MO, USA) and submerged within a starter buffer (25 mM NaH_2_PO_4_, 250 mM NaCl, 10 mM imidazole, pH 7.6). Dialysis tubing was retained at 4 °C in a 1:5 culture to buffer ratio until three buffer changes occurred with a minimum of 4 h per exchange. Tubing was then removed from buffer and covered in polyethylene glycol (PEG) 8000 powder at 4 °C until the dialysed supernatant volume reduced around four-fold.

Recombinant proteins were purified using nickel-nitrilotriacetic acid (Ni-NTA) affinity chromatography. Briefly, the concentrated culture supernatant was added to 1 mL of pre-equilibrated Ni-NTA agarose resin (QIAGEN, Hilden, Germany) under gravity pressure at 1 mL/min. The resin was then washed with 10 column volumes of starter buffer containing 20 mM imidazole, and bound proteins were eluted with starter buffer containing, firstly, 100 mM of imidazole, then, secondly, 400 mM of imidazole. Elutions were combined and buffer exchanged into 1× PBS (120 mM NaCl, 5 mM NaH_2_PO_4_·H_2_O, 16 mM Na_2_HPO_4_, pH 7.4) using Amicon^®^ Ultra-15 centrifugal filters units (Merck, Kenilworth, IL, USA) with a 3 kDa cut-off. The N-linked glycosylation structures on the FhTSP2 sequence were removed from rLTB-FhTSP2 and rFhTSP2 using Endoglycosidase H (Promega) at 500 mU/mL as per the manufacturer’s instructions. Protein concentrations were determined after deglycosylation using the Pierce BCA Protein Assay Kit (Thermo Fisher Scientific) according to the manufacturer’s instructions.

### 2.3. Protein Separation via SDS-PAGE and Western Blot Analysis

Samples after purification and deglycosylation were analysed by SDS-PAGE separation using a 4–12% gradient Mini-PROTEAN TGX^TM^ Pre-cast gel (Bio-Rad, California, USA) and stained with 0.1% (*w*/*v*) Coomassie brilliant blue R. Purified deglycosylated samples were mixed with 2× Laemmli sample buffer with the addition of 120 mM dithiothreitol (DTT). Samples of deglycosylated rLTB-FhTSP2 and rLTB were prepared in duplicate, with one sample boiled for 10 min at 95 °C and the other not boiled for assessment of oligomerisation (each in the presence of 120 mM DTT). Gels were replicated with one stained with Coomassie and the other used for Western blot analysis. Deglycosylated bands from boiled and non-boiled samples were excised from the gel and analysed for mass spectrometry sequencing (La Trobe Comprehensive Proteomics Platform, Melbourne, VIC, Australia).

For Western blot analysis, proteins were electrotransferred onto a PVDF membrane via the 7-min transfer protocol on the Trans-Blot^®^ Turbo™ System (Bio-Rad). Membranes were then blocked with blocking buffer A (5% (*w*/*v*) skim milk in TBS-T: 50 mM Tris-HCl (pH 7.6), 150 mM NaCl, 0.1% (*v*/*v*) Tween 20) and incubated for 1 h at room temp (RT) with orbital shaking. Subsequently, membranes were incubated with mouse anti-His tag horse radish peroxidase (HRP)-conjugated antibodies (R&D Systems, Minneapolis, MN, USA) diluted 1:10,000 in blocking buffer A for 1 h at RT with orbital shaking. Membranes were then washed three times with TBS-T prior to development with Clarity Western ECL™ substrate (Bio-Rad) and imaged on the C-DiGit^®^ Blot Scanner (LI-COR Biosciences, Lincoln, NE, USA).

### 2.4. Characterisation of Antigenicity of Recombinant Proteins

An enzyme-linked immunosorbent assay (ELISA) was used to determine FhTSP2 antigenicity within the rLTB-FhTSP2 fusion complex using a bovine immune serum obtained from a previous vaccine trial with rFhTSP2. Medium binding 96-well microtiter plates (Greiner Bio-one, Kremsmünster, Austria) were coated with equal molar concentrations of individual antigens (rFhTSP2 or rLTB) to equal the concentrations within the fusion protein (rLTB-FhTSP2): this was performed by adding 5 μg/mL of rFhTSP2, 9.8 μg/mL of rLTB-FhTSP2, and 5 μg/mL of rLTB diluted in coating buffer (28.6 mM Na_2_CO_3_, 71.5 mM NaHCO_3_, pH 9.6) at 100 μL/well and incubating overnight at 4 °C. Plates were subsequently washed three times with 1× PBS-T and blocked with 200 μL/well of blocking buffer B (5% skim milk in 1× PBS-T: PBS, 0.05% Tween 20) and incubated at 37 °C for 2 h. Pooled bovine sera from prior to vaccination (*n* = 3) and post-vaccination (*n* = 3) were diluted (1:100, 1:200, 1:400, 1:800) in blocking buffer B, and 100 μL was added to each well in triplicate and incubated for 1 h at 37 °C. Plates were then washed five times with 1× PBS-T and incubated with 100 μL/well of rabbit anti-bovine IgG HRP conjugate antibodies (Sigma-Aldrich) diluted 1:5000 in blocking buffer B for 1 h at 37 °C. The plates were washed five times with 1× PBS-T and developed with 100 μL/well of 3,3′,5,5′ tetramethylbenzidine (TMB) substrate (Life Technologies, Carlsbad, CA, USA). Plates were incubated in the dark at RT for 8 min, and development was stopped with 100 μL/well of 1 M HCl. The optical density (OD) was read at 450 nm using an xMark™ Microplate Spectrophotometer (Bio-Rad). Background serum control wells were set up with no antigen coated; these OD values were subtracted from the average OD of the antigen-coated wells to display the results as the adjusted OD.

### 2.5. Characterisation of GM1 Binding of Recombinant AB_5_ Toxin

LTB pentamer formation was determined by a GM1-ganglioside binding ELISA. Microtiter plates were coated (100 μL/well) with 3 μg/mL monosialoganglioside GM1 from bovine brain (Sigma-Aldrich) in a coating buffer along with coating-buffer-only control wells and incubated overnight at 4 °C. The plate was washed three times with 1× PBS-T and blocked with 200 μL/well of blocking buffer C (3% (*w*/*v*) bovine serum albumin (BSA) in 1× PBS-T) for 2 h at RT, then washed again three times with 1× PBS-T. Proteins (rEcxB; a positive GM1 binding control [46], rLTB-FhTSP2, and rFhTSP2) were incubated in 100 μL/well triplicates, at ten-fold serial dilutions (3, 0.3, and 0.03 μg/mL) in blocking buffer C for 2 h at RT. After washing five times with 1× PBS-T, plates were incubated for 1 h at RT with 100 μL/well of mouse anti-His tag HRP conjugate antibodies (R&D Systems) diluted 1:10,000 in blocking buffer C. Plates were washed five times with 1× PBS-T with the reactions developed and stopped and the plate OD read as described above. Background control wells were not coated with GM1: these OD values were subtracted from the average OD of the GM1-coated wells and presented as the adjusted OD. 

### 2.6. Experimental Animals and Immunisation

Angus and Angus Hereford cross female cattle 6 months of age were confirmed negative for *F. hepatica* infections through a liver fluke faecal egg count (FEC), the liver fluke coproantigen ELISA (BioX Diagnostics, Rochefort, Belgium), and the liver fluke serological ELISA (BioX Diagnostics), following the manufacturer’s instructions, as described [47]. Cattle were housed at CSIRO research station in Armidale, New South Wales, Australia, and were randomly allocated into groups of six with all groups containing animals with a similar average body weight (165.1, 165.5, 168.2 and kg for Groups 1, 2, and 3, respectively). For the duration of the study, animals had ad lib access to pasture and were kept on a single grazing paddock that did not contain waterbodies for the propagation of the intermediate snail host of liver fluke. 

All animals were injected on both Day 0 and Day 28, challenged with infective metacercaria on Day 42, and sacrificed on Day 125 or 126. As antigens were solubilised in PBS; the control group (Group 1) was subcutaneously immunised with 1 mL of PBS solution alone on either side of the lateral aspect of the neck at each time point. Vaccination Groups 2 and 3 received the same molar amount of the rFhTSP2 antigen. This equated to Group 2 intranasally receiving 1 mL of rLTB-FhTSP2 protein (451 µg) in PBS on both Day 0 and 28 and no additional adjuvant: animals were vaccinated in the left nostril on each occasion. Group 3 was subcutaneously immunised with 200 µg of rFhTSP2 in PBS emulsified within 1 mL of Freund’s complete adjuvant (Sigma-Aldrich) on Day 0 and 1 mL of Freund’s incomplete adjuvant (Sigma-Aldrich) on Day 28 on either the left (Day 0) or right (Day 28) side of the lateral aspect of the neck at each time point. All animals were orally challenged 6 weeks after the first immunisation (Day 42) with 350 viable metacercaria (Oberon strain, Invetus Pty Ltd., Armidale, Australia) and sacrificed 12 weeks post infection (Day 125 or 126, denoted as Day 125). Blood samples were taken to assess each immune response: pre-vaccination (Day 0), post-vaccination (Day 42), after the challenge (Day 84), and at the end of the trial (Day 125). Faecal samples were obtained as per [48] and collected before the start (day −22), on Day 42, and at the conclusion (Day 125) of the trial for FEC. Invetus Pty Ltd. processed the bloods and completed the FEC and necropsies. All animal welfare was conducted in accordance with the University of New England Animal Ethics Committee approval number AEC17-084 (Invetus study number AVBAB17039). The study was covered by APVMA small trial permit no. PER 7250 and Department of Agriculture and Water Resources in vivo permit #2018-053 (governing QC1 requirements for biological material use in non-laboratory animals).

### 2.7. Assessment of Protection

Animals were euthanised on Days 125 and 126 with their livers, gall bladders, and associated small intestine removed and collected as described [47]. All contents were removed and examined for liver flukes. The liver was sectioned into 0.5–1 cm strips, carefully squeezing and collecting flukes before a new section was cut. Any remaining flukes were collected after overnight incubation of the liver sections in warm PBS that was then sieved and rinsed thoroughly. The percentage of vaccine efficacy was determined using the equation:

$$\mathrm{Eficacy}=\frac{\left(\mathrm{control}\;\mathrm{average}\;\mathrm{fluke}\;\mathrm{burden}\;-\;\mathrm{vaccination}\;\mathrm{group}\;\mathrm{average}\;\mathrm{fluke}\;\mathrm{burden}\right)}{\mathrm{control}\;\mathrm{average}\;\mathrm{fluke}\;\mathrm{burden}}$$

### 2.8. Assessment of Antibody Responses

The antigen-specific humoral immune response of serum IgG, serum IgA, and saliva IgA towards rFhTSP2 was assessed by an in-house developed ELISA. Wells of a microtiter ELISA plate were coated with 100 μL/well of 5 μg/mL of rFhTSP2 in coating buffer in duplicate wells per sample and coating buffer only for background control wells. Plates were then incubated overnight at 4 °C. After three washes with 1× PBS-T, plates were blocked with 200 μL/well of blocking buffer B for 2 h at 37 °C. Subsequently, plates were washed as above and incubated in duplicate with 100 μL/well of serum (diluted 1:50) or saliva (diluted 1:8) within a blocking buffer at 37 °C for 1 h. Plates were washed three times with 1× PBS-T and incubated with a conjugate detection antibody. Serum was incubated with rabbit anti-bovine IgG HRP antibody (Sigma-Aldrich) diluted 1:5000 and rabbit anti-bovine IgA HRP antibody (Bethyl, Montgomery, TX, USA) diluted 1:2000, whereas saliva was only incubated with the IgA detection antibody. Plates were thoroughly washed three times with 1× PBS-T prior to the addition of TMB. The colour reactions developed and stopped, and OD read as above. Individual animal results are displayed as the adjusted OD, after the mean OD of the background control wells was subtracted from the average specific OD for each individual sample. 

The in-house ELISA assay was developed for an optimal saliva dilution using the above protocol by testing pooled (*n* = 6) saliva dilutions (1:2.5, 1:5, 1:10, 1:10, 1:40, and 1:80) pre- and post- vaccination with the secondary IgA detection antibody at varying dilutions (1:1000, 1:2000, 1:3000, and 1:6000). All assays had individual serum and saliva background controls (no antigen coated) in duplicate and secondary positive and negative controls in duplicate.

### 2.9. Statistical Analysis

Statistical analysis was performed using OriginPro 2020 Software. Any differences of means were analysed using a two-sample *t*-Test with the Welch correction. Spearman correlations were used to analyse relationships between the fluke burdens and wet weights and the humoral responses. *p* values ≤ 0.05 were considered statistically significant. 

## 3. Results

### 3.1. Recombinant LTB-TSP2 Fusion Protein Expressed in Pichia pastoris

**Figure 1:**
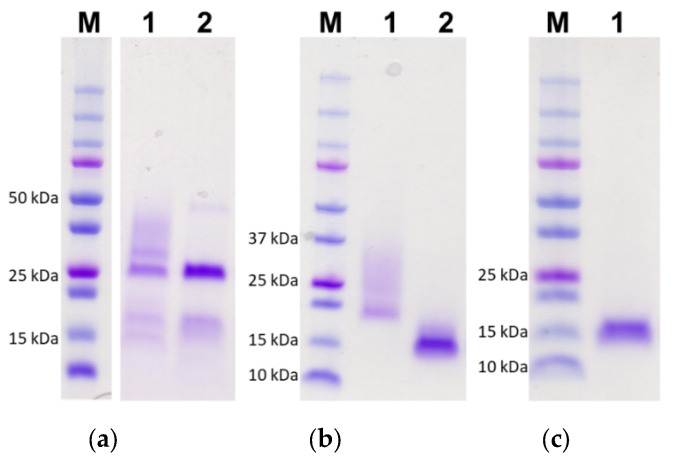
Recombinant expression and purification of rLTB-FhTSP2, rFhTSP2, and rLTB. Yeast culture supernatant for (**a**) rLTB-FhTSP2, (**b**) rFhTSP2, and (**c**) rLTB were each applied to a Ni-NTA column and washed before elution with starter buffer containing 100–400 mM imidazole. A total of 15 µL of each pooled and concentrated nickel elutions (lane 1) and deglycosylated rLTB-FhTSP2 and rFhTSP2 after incubation with Endoglycosidase H (lane 2) was resolved by SDS-PAGE and stained with Coomassie blue. M, molecular weight markers.

The DNA sequences encoding LTB-FhTSP2 and FhTSP2 were cloned into the pPICZαA vector, and recombinant rLTB-FhTSP2 and rFhTSP2 were expressed as secreted proteins from *P. pastoris* to act as vaccine antigens. Both rLTB-FhTSP2 and rFhTSP2 were successfully expressed after methanol induction and purified from X33 *P. pastoris* culture supernatants as shown by SDS-PAGE (Figure 1). The non-glycosylated rLTB appeared at its expected size of 14 kDa. Incubation of purified rLTB-FhTSP2 and rFhTSP2 with Endoglycosidase H removed carbohydrate groups from the ECL2 sequence of FhTSP2, which formed dense bands around the expected sizes of 22 kDa for rLTB-FhTSP2 and 14 kDa for rFhTSP2. The average yields of recombinant protein per yeast culture after purification were 4.4 mg/L (rLTB-FhTSP2, *n* = 4), 8.9 mg/L (rFhTSP2, *n* = 4), and 6.2 mg/L (rLTB, *n* = 2).

### 3.2. Characterisation of Oligomerisation and Antigenicity of rLTB-FhTSP2

**Figure 2:**
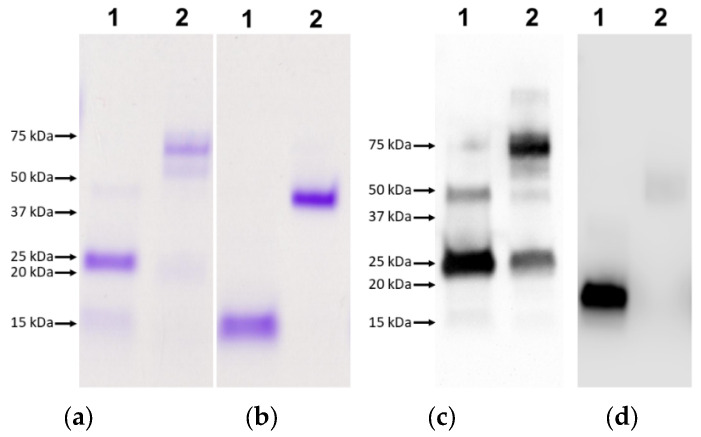
Characterisation of LTB oligomerisation. Both proteins rLTB-FhTSP2 (**a**,**c**) and rLTB (**b**,**d**) were loaded at 6 µg and subject to boiling at 95 °C for 10 min. Boiled (lane 1) and non-boiled (lane 2) proteins were analysied by SDS-PAGE and visualized by Coomassie staining (**a**,**b**) and Western blotting (**c**,**d**) using anti-His tag antibodies.

**Figure 3:**
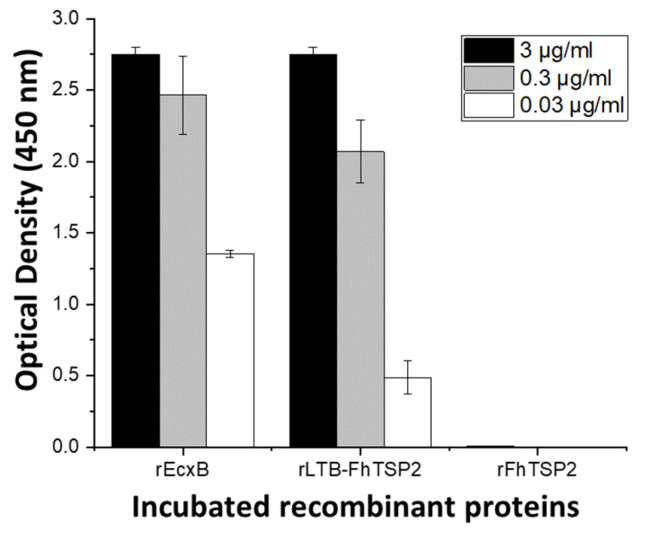
Characterisation of pentamer formation of the rLTB-FhTSP2 fusion complex via glycan receptor binding. Plates were coated with 3 μg/mL of GM1 ganglioside and blocked with 3% bovine serum albumin. Potential binding proteins (rEcxB and rLTB-FhTSP2) and negative control (rFhTSP2) were incubated in triplicate at three dilution factors (3, 0.3, and 0.03 μg/mL). Binding was detected by mouse anti-His tag HRP conjugated antibodies. All results are displayed as the mean of the adjusted optical density after subtraction of non-GM1 coated wells.

The adjuvant effect of LT is dependent on the toxin forming a pentamer structure [41]. The functional pentamer formation of recombinant rLTB-FhTSP2 fusion protein was shown by SDS stability and glycan binding. Several AB_5_ toxins have B subunit pentamers that are resistant to denaturation with SDS [49]. Analysis by SDS-PAGE confirmed SDS stability and oligomerisation of both rLTB and rLTB-FhTSP2, as without sample boiling, they appeared at approximately 45 and 70 kDa, respectively, thus suggesting that both rLTB and rLTB-FhTSP2 are assembled into pentamers and confirmed as recombinant proteins by an anti-His tag Western blot (Figure 2). Functional binding of rLTB-FhTSP2 to glycan GM1 was observed by ELISA (Figure 3). Different concentrations of rLTB-FhTSP2 bound to GM1 at similar levels relative to the EcxB toxin, which has previously been shown to strongly bind GM1 [46]. This indicates that the expressed rLTB-FhTSP2 forms a functional pentamer. Expressed rFhTSP2 alone did not bind to GM1 (Figure 3).

**Figure 4:**
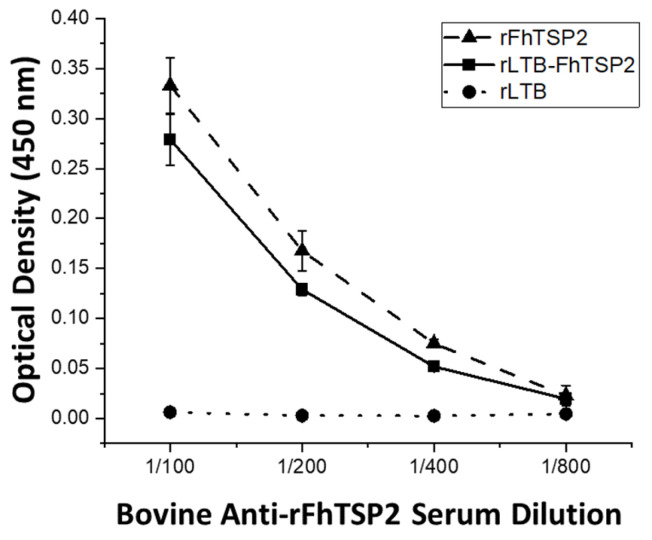
Characterisation of the antigenicity of the rLTB-FhTSP2 fusion complex. The reactivity of rFhTSP2, rLTB-FhTSP2, and rLTB was analysed using diluted bovine anti-rFhTSP2 sera (1:100, 1:200, 1:400, and 1:800) and a anti-bovine IgG HRP secondary antibody. Antigens were coated at an equal molar ratio of the FhTSP2 protein sequence.

To determine if the antigenicity of FhTSP2 is affected by fusion with the LTB protein, recombinant proteins were tested for antibody recognition against immune sera from cattle previously vaccinated with unfused rFhTSP2. Bovine sera prior to vaccination with rFhTSP2 did not show reactivity towards rLTB, rFhTSP2, or rLTB-FhTSP2 (Figure S2). The immune sera did not react with rLTB, while it did react with rFhTSP2 and rLTB-FhTSP2 at similar levels over a range of dilutions, suggesting the fusion protein retained antigenicity (Figure 4). The elevated reactivity of rFhTSP2 could have occurred from cows developing a response towards the *c-myc* epitope within rFhTSP2 and not within the rLTB-FhTSP2 sequence. 

### 3.3. Efficacy Assessment of rFhTSP2 and rLTB-FhTSP2 as Vaccines in Cattle

**Figure 5:**
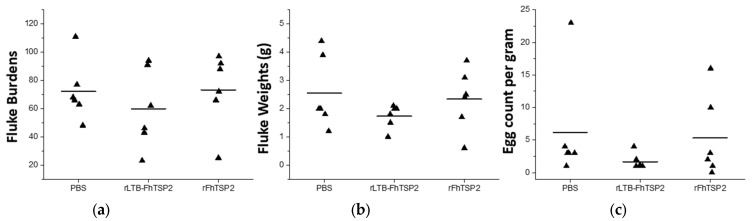
Liver fluke infection parameters for each vaccination group. (**a**) Liver fluke burdens, (**b**) fluke wet weights, and (**c**) faecal egg counts for each group. PBS: infection control vaccinated with PBS, rLTB-FhTSP2: intranasal vaccination with rLTB-FhTSP2; rFhTSP2: subcutaneous vaccination with rFhTSP2. Each animal is denoted as a black triangle and the mean fluke burden for each group is represented by a solid line.

The efficacy of rFhTSP2 and rLTB-FhTSP2 as vaccine candidates was assessed in cattle (Table 1, Figure 5). After infection with 350 metacercaria, the average mean fluke numbers for the control PBS vaccinated group was 72.2 ± 21.2. The rLTB-FhTSP2 intranasally vaccinated animals showed a mean fluke count of 59.8 ± 28.2, which was 17% lower than the control group, but this reduction was not significant. The mean for the rFhTSP2 subcutaneously vaccinated animals was 73.3 ± 26.5, indicating no protection. The rLTB-FhTSP2 group had 3/6 animals with fluke burdens lower than the lowest fluke burden (48) observed in the control group: 23, 43 and 46. The fluke wet weights in the control and rFhTSP2 vaccinated group had similar averages of 2.6 ± 1.3 g and 2.3 ± 1.1 g, respectively (Figure 5b). The rLTB-FhTSP2 group had a fluke wet weight average of 1.7 ± 0.4 g, and all recovered fluke wet weights were below the average mean of the control group. The FEC for the control and rFhTSP2 vaccinated group was similar with mean counts of 6.2 ± 8.3 epg and 5.8 ± 6.2 epg, respectively (Figure 5c). The observed FEC for the rLTB-FhTSP2 vaccinated animals was lower and had a tighter range at 1.7 ± 1.2 epg, showing a FEC reduction of 73%. However, neither the fluke burdens, fluke wet weights, or FEC for either vaccination group were significantly different to the control group.

### 3.4. Humoral IgG and IgA Responses in Cattle and Correlations with Fluke Burdens

**Figure 6:**
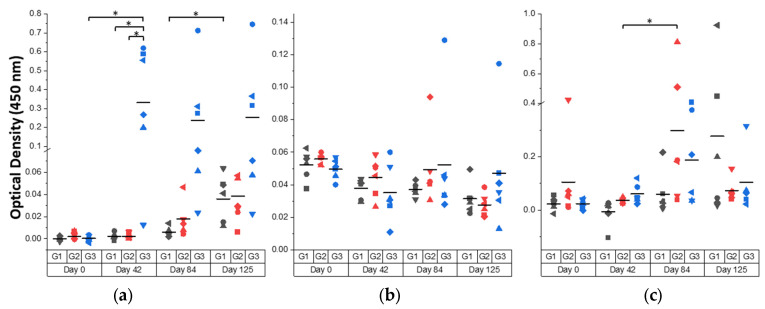
Bovine humoral response towards rFhTSP2 over the course of the trial. (**a**) Serum IgG, (**b**) serum IgA, and (**c**) saliva IgA responses from Day 0 (pre-vaccination), Day 42 (post-vaccination), Day 84 (post-challenge), and Day 125 (end of trial) from Group 1 (G1: dark grey) subcutaneously vaccinated with PBS; Group 2 (G2: red) intranasally vaccinated with rLTB-FhTSP2; and Group 3 (G3: blue) subcutaneously vaccinated with rFhTSP2. Each animal within a group is represented by a different shape. The reactivity of 5 μg/mL of rFhTSP2 was analysed using serum (diluted 1:50) or saliva (diluted 1:8) and an anti-bovine IgG HRP secondary antibody diluted 1:5000 or a anti-bovine IgA HRP antibody diluted 1:2000. The mean OD of each group is represented by a solid line. Significant differences (*p* < 0.05) of the mean are denoted by asterisks (*).

Vaccination with rFhTSP2 and rLTB-FhTSP2 induced a range of antigen-specific serum IgG, serum IgA, and mucosal IgA responses in cattle. The humoral response kinetics against vaccine antigen rFhTSP2 were assessed in all animals using serum and saliva samples (Figure 6). Animals subcutaneously vaccinated with rFhTSP2 showed the strongest serum IgG response during the trial, and a significant increase in serum IgG after vaccination (Day 42) and throughout the infection period was observed in the rFhTSP2 vaccinated group. There was a weak specific serum IgG response in animals following rLTB-FhTSP2 intranasal vaccination with no significant difference in antibody levels compared to the control. In general, there was an unsubstantial antigen-specific serum IgA response generated in all groups across the trial. Contrastingly, a rise in the saliva IgA response was observed in both vaccination groups at Day 84 and in the infection control group at Day 125, with the average mucosal IgA response in the rLTB-FhTSP2 vaccination group significantly higher at Day 84 compaired to its prior time point. Overall, the humoral responses gained in this trial were largely individual-animal-based with large differences between animals in anti-FhTSP2 IgG and IgA levels in each group.

**Figure 7:**
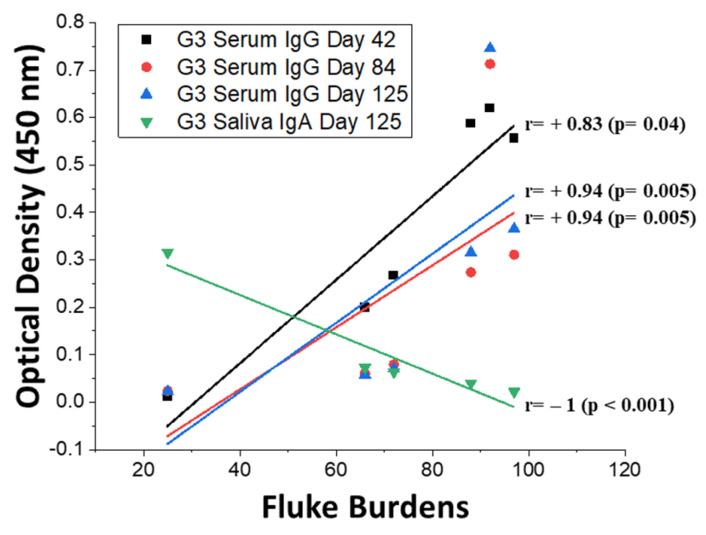
Spearman correlations between bovine humoral responses and the final liver fluke burdens in Group 3. Significant positive relationships were noted between fluke burdens and serum IgG at Day 42 (black: r = +0.83, *p* = 0.04), Day 84 (red: r = +0.94, *p* = 0.005), and Day 125 (blue: r = +0.94, *p* = 0.005), for Group 3 (G3) subcutaneously vaccinated with rFhTSP2. A significant negative relationship was seen for G3 between fluke burdens and saliva IgA at Day 125 (green: r = −1, *p* < 0.001). No significant correlations were seen between fluke burdens and serum or saliva responses for Group 2 after rLTB-FhTSP2 vaccination (Table S1). Linear regression lines display the significant relationships between antibody enzyme-linked immunosorbent assay optical density and the individual fluke burden.

Analysis of the associations between antibody responses of each animal and their fluke burdens determined that there was a significant positive relationship between fluke burdens and the serum IgG responses in Group 3 after subcutaneous rFhTSP2 vaccination and throughout the trial (Day 42: r = +0.83, *p* = 0.04; Day 84: r = +0.94, *p* = 0.005; Day 125: r = +0.94, *p* = 0.005) (Figure 7). Conversely, the anti-rFhTSP2 saliva IgA responses in Group 3 at Day 125 were significantly negatively associated with the final fluke burdens (r = −1, *p* = < 0.001). No apparent correlations were evident in the intranasal rLTB-FhTSP2 group for serum IgG or saliva IgA (Table S1). Antibody levels determined in this trial seemingly did not suppress the liver fluke burdens. 

## 4. Discussion

There is an urgent requirement to develop an efficacious *F. hepatica* vaccine to control the parasite due to the emergence of drug-resistant fluke populations that dampen current control measures [7,8]. Many studies have aimed to use vaccines to stimulate a protective immune response in cattle with varying degrees of success [2,9]. We hereby report the use of the recombinant tegumental antigen (rFhTSP2) and FhTSP2 linked to a mucosal protein adjuvant (rLTB-FhTSP2) as vaccine candidates in cattle.

Tetraspanins are key components of the trematode tegument layer, playing crucial roles in tegument formation and rigidity as mRNA silencing of TSP2 and TSP3 in both *O. viverrini* and *S. mansoni* resulted in a thinner impaired tegument [50,51]. In addition, TSP2 from *O. viverrini* and *S. mansoni* were both expressed on the tegument and exposed to the host immune system [25,51]. Vaccination with membrane-bound and surface-exposed vaccine antigens could increase the effectiveness of cytotoxic killing of liver fluke via ADCC, which juvenile flukes are susceptible towards in vitro [13,52,53]. ADCC is the process of granular attack from host effector cells that are activated after antibodies have bound to antigens on the fluke surface [35,52,53]. As a proposed defence mechanism, juvenile flukes repeatedly remove or slough their outer tegument layer, essentially removing bound immunoglobulins and rendering cellular granulation less effective [54,55,56]. Therefore, an approach to vaccinology against liver fluke would be to generate high levels of immunoglobulins towards a tegument surface protein that initiates effective fluke killing to ultimately outperform the sloughing evasion mechanism. 

*F. hepatica* tetraspanin TSP2 (ECL2 domain) generated in a yeast-recombinant expression system and formulated in Freund’s adjuvant was unable to elicit protection to any degree in cattle after subcutaneous vaccination. This was unexpected as recombinant SmTSP2 has been evaluated as a vaccine against schistosomiasis in vaccine trials and gained partial protection in small animal models [25,26,27]. With *S. mansoni*, rSmTSP2 was presented to mice fused to *E. coli* thioredoxin and induced a mean efficacy of 57% across two trials in mice [25]. The single antigen efficacy in mice was then improved after fusion with the immunogenic 5B fragment of the hookworm aspartic protease with a 29–31% increase in efficacy with the rSmTSP2/5B compared to rSmTSP2 alone [26]. In addition, rSm29 antigenic protection was improved in two rSmTSP2/Sm29 chimeric forms that linked SmTSP2 to either the N- or C- terminus of Sm29, inducing 28% or 35% efficacy, respectively, in mice [27]. While the *S. mansoni* TSP2 antigen elicits protection with an indication for optimisation, the TSP2 antigen family from *S. japonicum* is polymorphic in nature and consists of at least seven different sequences [57]. One SjTSP2 sequence was able to induce significant protection in mice (46–58%), while two other sequence variants were unable to evoke consistent protection in mice [57,58,59]. The *F. hepatica* genome also displays a high level of sequence polymorphism, potentially due to the parasite adapting to multiple hosts and surviving in different global climates [16]. Therefore, it could be invaluable to examine the diversity of TSPs among different *F. hepatica* isolates to determine the potential of TSPs as vaccine antigens and understand the allelic diversity of TSP2 in parasite populations recovered in this study, which could explain the lack of efficacy observed using TSP2 in this study. However, exploration into allelic variability determined that there was a low level of sequence polymorphism in lead *F. hepatica* vaccine candidates, including CL1, suggesting that sequence polymorphism is unlikely to have contributed to the previously observed variations in vaccine efficacy [9,12,33,60]. 

Prior to vaccination, the expressed rLTB-FhTSP2 fusion protein was determined as being antigenic in cattle and biologically active as it bound to the GM1 glycan ligand. When intranasally delivered to the mucosal surface in cattle, rLTB-FhTSP2 produced 17% efficacy (non-significant) in the reduction of fluke burden and a (non-significant) 73% reduction of FEC in comparison to subcutaneous rFhTSP2 vaccination, which showed no efficacy or significant reduction of FEC relative to the control group. Needle-free mucosal delivery is a potentially safer alternative to traditional vaccine administration and may be preferable for a vaccine against a pathogen that resides or establishes at the mucosal surface, as it can beneficially induce both mucosal and systemic immunity in addition to making the vaccine deployment easier [61,62]. In mice models, mucosal administration of LTB has been shown to significantly increase IFN-γ levels, initiating a Th1-like immune bias and increasing specific serum and mucosal humoral responses [63,64,65]. LTB improves immunogenicity to fused antigens by increasing antigen processing and presentation to APCs, stimulating a humoral response through activating B and T cells, and can influence T cell differentiation and the subsequent cytokine induction [38,40,66]. There has been limited investigation into the immunomodulatory properties of the AB_5_ toxins in livestock. Vaccination of pigs with LTB has been shown to elevate the specific IgG and IgA responses towards three fused *Mycoplasma hyopneumoniae* antigens and the GP5 epitope of porcine reproductive and respiratory syndrome virus after mucosal vaccination [42,67]. Additionally, both pigs and cattle had enhanced responses to previously poorly immunogenic antigens after being combined with a mutant LT and delivered intranasally [68]. Pigs showed high levels of specific mucosal IgA and serum IgG and were protected from challenge with highly virulent strains of *Erysipelothrix rhusiopathiae* and *Bordetella bronchiseptica* [68]. Cattle vaccinated with the adhesin antigen intimin, of enterohemorrhagic *E. coli*, showed elevated levels of specific IgG in serum and colostrum and elevated specific IgA in nasal and saliva samples towards intimin [68]. Furthermore, CTB and LTB fusions have been analysed as vaccines through intranasal administration in cattle and were shown to stimulate antibodies and enhanced resistance towards *Mannheimia haemolytica* and *Clostridium botulinum*, respectively [69,70]. 

The approach to liver fluke vaccinology by generating mucosal immunity aims to target NEJ flukes before infection causes liver damage and thus limit production decline. When ITT sheep display natural resistance to *F. gigantica*, they are capable of initiating an immune-mediated parasite killing within 2 weeks of infection [71]. Furthermore, vaccine studies show that when protection is gained, the hosts (sheep, cattle, and goats) show reduced hepatic damage, indicating that flukes are killed early, likely while they reside at the host’s mucosal surface or peritoneal cavity [71,72,73,74]. Mucosal vaccination has shown promise against liver fluke infections using cysteine protease (FhCP) or cathepsins (FhCat) as the antigen. Notably, after oral administration with transgenic freeze-dried lettuce expressing FhCP fused to a carrier molecule, hepatitis B core protein, both calves (56.2%, *p* < 0.05) and lambs (35.5%, *p* > 0.05) had reduced *F. hepatica* burdens despite a lack of specific serum IgA induction [75]. Additionally, FhCP in the form of inclusion bodies from *E. coli* induced 54.2% (*p* < 0.01) protection in female calves after intranasal administration, and when analysed through different administration routes in lambs, males showed higher protection after intranasal administration (56.5%, *p* < 0.01) compared to intramuscular administration (30.1%, *p* > 0.05) [76]. Moreover, when sheep were co-administered with FhCatL5 and FhCatB2 intranasally formulated with the immune stimulating complexes (ISCOMs) containing QuilA and CpG-ODN 2135 or intramuscularly with QuilA, only the intranasal group induced significant protection of 40.5% (*p* = 0.006) compared to 20.9% (*p* > 0.05) in the intramuscular group [77]. Similar to the present study, a greater specific antibody response was noted in the intramuscular group that did not confer protection [77]. Ultimately, mucosal vaccination targeting NEJs warrants further investigations as it has the potential to combat liver fluke infections.

## 5. Conclusions

From the present study, we can deduce that intranasal delivery of chimeric rLTB-FhTSP2 in cattle enhanced antigen-specific mucosal IgA after a liver fluke challenge to a greater capacity than subcutaneous delivery of rFhTSP2 in Freund’s adjuvant, but unlike some of the aforementioned studies, it did not induce a strong IgG response towards the linked antigen. Unfortunately, the rFhTSP2 antigen formulated with Freund’s adjuvant was unable to generate any protection, despite generating a strong serum IgG response. It appears that rFhTSP2 can be discounted as a vaccine candidate under the vaccination conditions used here; however, further investigation of FhTSP2 as a vaccine antigen with different adjuvants is warranted, as it is an orthologue to the partially protective SmTSP2 and OvTSP2 antigens. The potential for LTB to boost the protection elicited against fused rFhTSP2 after the parasite challenge did not effectively determine LTB’s adjuvant affects, and linking to a known immune driving antigen is required for proper assessment. Perhaps testing various adjuvants, especially mucosal adjuvants, should be at the forefront of liver fluke vaccinology to generate a response that kills the parasites at the site of entry and helps farmers experience on-farm production benefits.

## Figures and Tables

**Table 1:** Final fluke infection parameters for the control and vaccination groups. Recovered fluke burdens, fluke wet weights, fecal egg counts, and host liver pathology values for animals within Group 1 (subcutaneous PBS vaccination), Group 2 (intranasal rLTB-FhTSP2 vaccination), and Group 3 (subcutaneous rFhTSP2 vaccination).

Group	Treatment (Delivery)	Fluke Burdens (mean ± SD)	Efficacay (% Protection)	Fluke Wet Weight (mean ± SD)	Fecal Egg Count (mean ± SD)	Egg Count Reduction	Liver Pathology Score (mean ± SD)
1	Control PBS (sc)	48, 63, 66, 68, 77, 111 (72.2 ± 21.2)	-	1.2, 1.8, 2, 2, 3.9, 4.4 (2.6 ± 1.3)	1, 3, 3, 3, 4, 23 (6.2 ± 8.3)	-	1, 2, 2, 2, 2, 3 (2 ± 0.6)
2	rLTB-FhTSP2 (in)	23, 43, 46, 62, 91, 94 (59.8 ± 28.2)	17%	0.6, 1, 1.5, 2, 2, 2.1 (1.7 ± 0.4)	1, 1, 1, 1, 2, 4 (1.7 ± 1.2)	73%	1, 2, 2, 2, 2, 2 (1.8 ± 0.4)
3	rFhTSP2 (sc)	25, 66, 72, 88, 92, 97 (73.3 ± 26.5)	−1.6%	0.6, 1.7, 2.4, 2.5, 3.1, 3.7 (2.3 ± 1.1)	0, 1, 2, 6, 10, 16 (5.8 ± 6.2)	5%	1, 2, 2, 2, 2, 2 (1.8 ± 0.4)

## Data Availability

The raw data presented in Figure 1 and Figure 2 in this study are openly available in FigShare at 10.26181/61074c202685b. Other data presented in this study are available in in the Supplementary Material within the article.

## Supplementary Materials

The following are available online at https://www.mdpi.com/article/10.3390/vaccines9111213/s1: Supplementary Data S1: The nucleotides translating TSP2 amino acid sequence (accession number Fh47871), Figure S1: Graphical representation of recombinant protein constructs within secretory vector pPICZα, Figure S2: Naïve sera control for characterisation of antigenicity of rLTB-FhTSP2 fusion complex. Table S1: Spearman correlation value between fluke burdens and bovine humoral responses in groups vaccinated with rFhTSP2 and rLTB-FhTSP2.
