# Supplementary material for: Evaluation of Immunogenicity and Efficacy of Fasciola hepatica Tetraspanin 2 (TSP2) Fused to E. coli Heat-Labile Enterotoxin B Subunit LTB Adjuvant Following Intranasal Vaccination of Cattle
Source: Vaccines (Basel). 2021 Oct 20;9(11):1213. doi: 10.3390/vaccines9111213 (PMC8623123; doi:10.3390/vaccines9111213)
Supplement: Supplementary file 1 [file vaccines-09-01213-s001.zip › vaccines-1374281-supplementary.pdf]

Data S1. The nucleotides translating TSP2 amino acid sequence (accession number Fh47871).

>FhD47871

atggcttccttaagttgtggttacaaatgtctccaggtgatcctggttattatgaatatcattgtggctatttgtgggatagcactgatcgtcgttggatca
attgcagaagttaactttaagaaatatgggacttcggatgacgtctatcttagagcattcgtaatcttcataatagcttttgggtgcttcatcgcgcttgtg
ggcatatttggcttctgtggagcttgtaaaaagagcgtttattgtcttaccttgtacattatcttcctttcaatcctcatacttgctggtttggctgccggtatt
gctggtttcgttctgaaggacaaagtcaaagagtacgtgacgaaaagcatgacagaagccttcaacaaatatcaagaccctacataccaaaaagtt
atcgataccattcagaaagacctcaaatgctgtggccccaacgggacgtggacaggttcgggaagtccgccaccttcatgctacggccctgatgga
cagctttactccgacggctgcgtacaaaatgttcaacaattcttcaagaaaaacgtagtgatcgtggccgcatgtgtctttggatttgcaattatccaaa
tactcggaattgtatttgctgcttgcgtttgtcaagcaatcaaaagaggtgaaacggcctaa

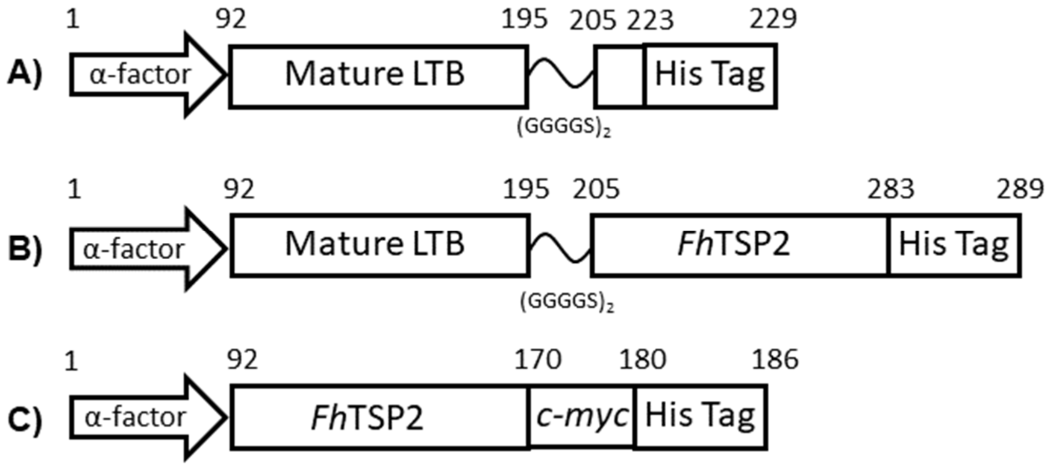

Figure S1. Graphical representation of recombinant protein constructs within secretory vector pPICZα. A) rLTB: plasmid α-factor secretory signal sequence upstream of the mature LTB sequence linked to a polyhistidine-tag via a flexible glycine rich linker $(GGGGS)_2$ and vector translated amino acids. B) rLTB-FhTSP2: plasmid α-factor secretory signal sequence upstream of the mature LTB sequence linked via $(GGGGS)_2$ to the extracellular loop 2 of the FhTSP2 sequence and a polyhistidine-tag. C) rFhTSP2: the plasmid α-factor secretory signal sequence upstream of the extracellular loop 2 of the FhTSP2 followed by the vectors c-myc epitope and polyhistidine-tag. Mature LTB, human variant (accession M178974) and recombinant FhTSP2 consists of the extracellular loop 2 from amino acid position 108 to 185 of FhD #47871.

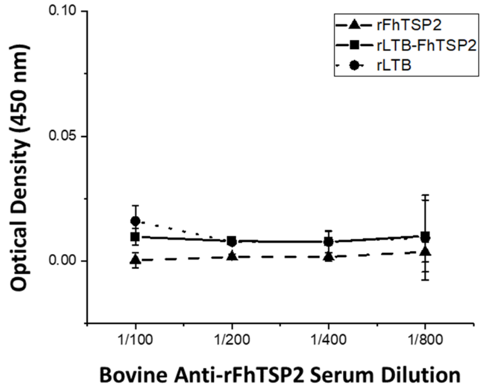

Figure S2. Naïve sera control for characterisation of antigenicity of rLTB-FhTSP2 fusion complex. Pooled (n=3) sera from cattle prior to vaccination with rFhTSP2 were used to test the reactivity of rFhTSP2, rLTB-FhTSP2 and rLTB using diluted bovine sera (1:100, 1:200, 1:400 and 1:800).

Table S1. Spearman correlation values between fluke burdens and bovine humoral responses in groups vaccinated with rFhTSP2 and rLTB-FhTSP2. Significant values ($p < 0.05$) are denoted with an asterisk (*).

	**Serum IgG**			**Serum IgA**			**Saliva IgA**		
	D42	D84	D125	D42	D84	D125	D42	D84	D125
rTSP2	0.06	−0.41	−0.26	−0.43	−0.46	−0.37	−0.06	0.09	0.09
(*p*-value)	(0.91)	(0.42)	(0.62)	(0.40)	(0.32)	(0.47)	(0.91)	(0.87)	(0.87)
rLTB-TSP2	0.83	0.94	0.94	−0.09	0.49	0.31	0.77	0.54	−1
(*p*-value)	(0.04) *	(0.005) *	(0.005) *	(0.87)	(0.32)	(0.54)	(0.07)	(0.27)	(<0.001) *
